# Opportunities, challenges and concerns for the implementation and uptake of pelvic floor muscle assessment and exercises during the childbearing years: protocol for a critical interpretive synthesis

**DOI:** 10.1186/s13643-017-0420-z

**Published:** 2017-01-25

**Authors:** Victoria E. Salmon, E. Jean C. Hay-Smith, Rachel Jarvie, Sarah Dean, Eivor Oborn, Susan E. Bayliss, Debra Bick, Clare Davenport, Khaled M. Ismail, Christine MacArthur, Mark Pearson, on behalf of the APPEAL study

**Affiliations:** 1 NIHR Collaboration for Leadership in Applied Health Research & Care South West Peninsula (PenCLAHRC), Institute of Health Research, University of Exeter Medical School, South Cloisters, St. Luke’s Campus, Heavitree Road, Exeter, EX1 2LU UK; 2 University of Otago, Dunedin, New Zealand; 3 Warwick Business School, University of Warwick, Coventry, UK; 4 University of Birmingham, Birmingham, UK; 5 King’s College London, London, UK

**Keywords:** Critical interpretive synthesis, Pelvic floor muscle exercise, Pelvic floor muscle training, Urinary incontinence, Midwifery practice, Implementation, Pregnancy, Postpartum, Antenatal education, Maternity services

## Abstract

**Background:**

Pregnancy and childbirth are important risk factors for urinary incontinence (UI) in women. Pelvic floor muscle exercises (PFME) are effective for prevention of UI. Guidelines for the management of UI recommend offering pelvic floor muscle training (PFMT) to women during their first pregnancy as a preventive strategy. The objective of this review is to understand the relationships between individual, professional, inter-professional and organisational opportunities, challenges and concerns that could be essential to maximise the impact of PFMT during childbearing years and to effect the required behaviour change.

**Methods:**

Following systematic searches to identify sources for inclusion, we shall use a critical interpretive synthesis (CIS) approach to produce a conceptual model, mapping the relationships between individual, professional, inter-professional and organisational factors and the implementation, acceptability and uptake of PFME education, assessment and training during the childbearing years. Purposive sampling will be used to identify potentially relevant material relating to topics or areas of interest which emerge as the review progresses. A wide range of empirical and non-empirical sources will be eligible for inclusion to encompass the breadth of relevant individual, professional, inter-professional and organisational issues relating to PFME during childbearing years. Data analysis and synthesis will identify key themes, concepts, connections and relationships between these themes. Findings will be interpreted in relation to existing frameworks of implementation, attitudes and beliefs of individuals and behaviour change. We will collate examples to illustrate relationships expressed in the conceptual model and identify potential links between the model and drivers for change.

**Discussion:**

The CIS review findings and resulting conceptual model will illustrate relationships between factors that might affect the implementation, acceptability and uptake of PFME education, assessment and training during the childbearing years. The model will inform the development and evaluation of a training package to support midwives with implementation and delivery of effective PFME during the antenatal period. The review forms part of the first phase of the United Kingdom National Institute for Health Research funded ‘Antenatal Preventative Pelvic floor Exercises And Localisation (APPEAL)’ programme (grant number: RP-PG-0514-20002) to prevent poor health linked to pregnancy and childbirth-related UI.

**Systematic review registration:**

PROSPERO: CRD42016042792

**Electronic supplementary material:**

The online version of this article (doi:10.1186/s13643-017-0420-z) contains supplementary material, which is available to authorized users.

## Background

Pregnancy and childbirth are important risk factors for urinary incontinence (UI) in women [1]. Prevalence rates of UI at 30 weeks gestation have been reported as 31% in nulliparous women and 42% in parous women [2]. Postpartum prevalence rates range from 30% in the first 3 months to up to 47% in the first 12 months postpartum [3]. It is reported that between two thirds and three quarters of women may still experience UI symptoms 12 years after delivery [4]. Incontinence places a large burden on women’s health and impacts on physical, mental and social quality of life [5], with associated pressure on healthcare resources and wider societal costs [6].

A Cochrane review investigated the effectiveness of pelvic floor muscle training (PFMT) (see Table 1 for a glossary of terms used in this review) for prevention and treatment of UI and faecal incontinence in pregnant and postnatal women [7]. The systematic review included 22 trials involving 8485 women and analysed the data according to whether PFMT interventions were for prevention of UI (pregnant women without prior UI) or for treatment (postnatal women symptomatic of UI) or were mixed prevention/treatment trials. The main findings were that, in prevention trials, women in their first pregnancy without prior UI who were randomised to PFMT and supervision were 30% less likely than women randomised to no PFMT or usual antenatal care to report UI up to 6 months after delivery [7].

**Table 1:** Glossary of pelvic floor muscle related terminology used in this review

Pelvic floor muscle contraction (PFMC)	PFMC refers to voluntary activation of the pelvic floor muscle (PFM). Correct contraction involves an inward lift and squeeze around the urethra, vagina and anus [10]
Pelvic floor muscle exercise (PFME)	This refers to performance of correct repeated PFMCs. A programme of repeated contractions is the basis of pelvic floor muscle training (PFMT)
Routine recommendation of PFME	This refers to recommendation of PFME to every pregnant woman as part of regular antenatal clinical practice. This might or might not be accompanied by further PFME education, assessment and/or training
PFME education	PFME education is the provision of information with the aim of increasing knowledge or understanding of PFME. This might include information about PFMC, how to perform a correct voluntary PFMC, why PFMT might be important during pregnancy, for example, to prevent or treat pelvic floor problems like urinary incontinence
PFMC assessment	This refers to subjective or objective assessment, or measurement, of PFM function, defined as the ability to perform a correct voluntary PFMC, and/or PFME, including the number of repetitions, and the quality and duration of contractions
Pelvic floor muscle training (PFMT)	PFMT refers to participation in a planned, progressive, supervised PFME programme to achieve a performance goal. Training involves teaching performance of a correct PFMC, tailored/individualised prescription of sufficient exercise dose (frequency, intensity, duration) to achieve desired changes in muscle physiology (for example, hypertrophy) and support for adherence to the prescribed treatment [32]
Implementation	In this review, implementation refers to the process of putting PFME education, assessment or PFMT into clinical practice
Uptake	An attempt made by a person to initially engage in an activity such as PFMT
Adoption	Regular performance of an activity in the short term
Maintenance	Sustained performance of an activity over time, including starting again after stopping (relapse or setback management)

UK guidelines for the management of UI in women recommend offering PFMT to women during their first pregnancy as a preventive strategy for UI (National Institute for Health and Care Excellence (NICE) [8]). NICE guidelines for antenatal care in uncomplicated pregnancies recommend providing information about pelvic floor muscle exercises (PFME) at the first appointment with a midwife, with an opportunity for women to ask questions and discuss the topic [9]. However, it has been suggested that in order for antenatal PFME to be effective, it should be delivered through a structured training programme (that is, ensuring PFME is performed correctly and regularly) [10] since information provision alone is seldom enough to support long-term (exercise) behaviour change [11].

Standardised terminology of pelvic floor muscle (PFM) function presented by the International Continence Society [12] (p.375) states that a correct pelvic floor muscle contraction (PFMC) should result in ‘a ventral and cranial movement of the perineum, and an upward movement of the pelvic organs together with an anterior movement caused primarily by the vaginal and rectal parts of the levator ani’. In other words, an inward lift and squeeze around the urethra, vagina and anus should occur [10]. An investigation into knowledge and performance of PFME in women attending a routine gynaecology appointment, without symptoms of PFM dysfunction, found that most women (94/120; 77%) had heard about PFME. However, approximately a third of these women were unable to perform an adequate PFMC on examination [13]. Lack of confidence with performing correct PFMC was also reported by a third (*n* = 720) of postpartum women in another study when asked about knowledge, practice and intention regarding PFME [14]. Therefore, assessment of PFMC may be important to ensure correct technique and obtain optimal outcomes from training.

To maximise the impact and effect the required change in PFME behaviour, it is essential to understand the individual, professional, inter-professional and organisational opportunities, challenges and concerns regarding implementation of PFME education, assessment and training during the childbearing years. Based on the authors’ previous experience with developing a national training programme to support implementation by midwives and obstetricians of evidence-based assessment and repair of perineal trauma (the PEARLS study, [15, 16]), it is recognised that understanding the context of care is essential to overcome barriers to support and sustain implementation.

It is anticipated that implementation in clinical practice, overall acceptability, uptake, adoption and maintenance (see glossary of terms, Table 1) of PFMT by women will be affected by the perceptions of individual healthcare professionals (HCPs) and women regarding PFME education, assessment and training. These are likely to be influenced by contextual factors such as organisational leadership (for example, how services are delivered and organised, including changes in service provision over time, funding models, professional organisation position statements, national guidelines, national policy), professional cultures and inter-professional work (for example, between midwives, physiotherapists and obstetricians) and social and cultural beliefs of women. We shall conduct a critical interpretive synthesis (CIS) [17] to identify interactions between these factors to explain how PFME can be implemented into midwifery practice in a way that introduce, enhance and/or sustain women’s ability to perform PFMT effectively.

## The ‘Antenatal Preventative Pelvic floor Exercises And Localisation (APPEAL)’ study

This CIS review forms part of the first phase of a United Kingdom National Institute for Health Research (NIHR) Programme Grants for Applied Research project entitled ‘Antenatal Preventative Pelvic floor Exercises And Localisation (APPEAL)’ to prevent poor health linked to pregnancy and childbirth-related UI. The overarching aim of APPEAL is to increase the number of women doing PFMT during pregnancy and to ensure the PFMC is correct and sufficient exercise is done to reduce the number of women who suffer symptoms of UI after giving birth. APPEAL will develop and test a training package for midwives. This training package will enable midwives to support pregnant women to perform a correct PFMC and encourage and supervise women doing PFMT. The APPEAL programme is designed to facilitate implementation of antenatal PFMT into routine practice by providing the missing elements required to effect an improvement change, in line with Batalden and Davidoff’s health service implementation framework [18].

The aim of the first phase of the APPEAL programme is to provide context awareness (Knowledge System 2 of the Batalden and Davidoff’s framework [18]) to improve implementation of evidence in health service delivery. This will be initially derived from two studies: (1) the CIS review, which will be complemented by (2) an ethnographic study to identify why in the context of organisational practices and cultural norms, midwives and other healthcare professionals and women behave the way they do regarding PFM assessment and exercises. A third study, a diagnostic test accuracy (DTA) systematic review (protocol currently awaiting registration with Cochrane), is also being conducted to identify the most accurate and acceptable tests (contributing to Knowledge System 3 of Batalden and Davidoff’s framework (performance measurement) [18]), performed by either practitioners or women to ensure correct PFMC and performance of PFME. The available tests identified in the DTA review will be mapped to findings regarding acceptability and feasibility of PFME assessment identified in the CIS and ethnography studies. Findings together will inform the identification, construction and development of conceptual categories to underpin a model showing relationships between factors likely to affect the implementation, acceptability, uptake and maintenance of PFME education, assessment and training during the childbearing years. This model will inform the development and evaluation of the APPEAL training package for midwives.

### Review objectives

The objectives of this review are:

1. To gain an understanding of:
  - Attitudes and beliefs of women about the positive or negative physical, social and psychological consequences of pregnancy and childbirth on their pelvic floor
  - Attitudes and beliefs of HCPs about women’s understanding of their pelvic floor and how they explain the function of the pelvic floor to women
  - Views of HCPs regarding the impact on women’s health of PFME education, assessment and training during the childbearing years
  - Women’s and HCPs’ views of organisational and/or professional issues and their understanding of how these impact on implementation, acceptability, uptake, adoption and maintenance of PFME education, assessment and training in the childbearing years
2. To develop a conceptual model that maps the relationships between individual, professional, inter-professional and perceived organisational factors and the implementation, acceptability and uptake of PFME education, assessment and training during the childbearing years.
3. To collate meaningful examples, either empirical or hypothetical, that illustrate the relationships expressed in the conceptual model.
4. To identify potential links between the conceptual model and drivers for change.

## Methods

The review will use CIS [17]. This approach has been chosen as it focuses on theory generation and will enable identification, construction and development of conceptual categories to underpin a conceptual model to inform future research in the APPEAL programme. More traditional systematic review methods take an aggregative approach to analysis, seeking to pool and summarise data from empirical studies, whereas CIS draws on qualitative research methods to synthesise a broad range of empirical and non-empirical evidence using an interpretive analysis. The synthesis of both quantitative and qualitative research is an advantage of CIS over other interpretive synthesis approaches, such as meta-ethnographic methods, as these have been limited to synthesis of qualitative research only [17]. The key processes in CIS are summarised in Table 2. We shall follow a transparent and systematic review process that allows for iterative development of the review question, inclusion criteria and purposive sampling methods and draws upon qualitative methods of inquiry [19].

**Table 2:** Summary of key processes in critical interpretive synthesis

Reflexivity	Constant reflexivity on the part of the review authors is essential to the critical interpretive synthesis (CIS) process, to ensure thorough searching and selection processes and generation of theory which is critically informed and credible in light of the evidence available
Review question	A review question should be proposed but left open to adjustment over the course of the research
Literature searching	The initial search strategy should be broad to identify potentially relevant papers close to the topic of interest. Searching, sampling, critique and analysis occur simultaneously
Purposive sampling	Purposive sampling of potentially relevant material is used to identify material which may fall outside of the initial search boundaries
Data extraction	Data extraction may be guided by formal data extraction procedures, but this is not essential for the CIS approach
Quality appraisal	Appraisal of the evidence aims to prioritise relevance and theoretical contribution to the review objectives, through critical interrogation of the evidence. Formal methodological quality appraisal of individual studies may be appropriate, but papers are not usually excluded on the basis of quality alone
Data analysis	Analysis aims to generate a synthesising argument or conceptual framework. This is developed through a critically informed synthesis of evidence included in the review. The framework should illustrate connections and relationships between new (synthetic) and existing constructs identified in the literature. The developing conceptual framework guides further selection of potentially relevant literature

### Reflexivity

Reflexivity is a key consideration in qualitative research [20]. The review team have drawn on the following definition by Mays and Pope (p.51) [20] to inform the reflexive accounting strategy in this study:

Reflexivity means sensitivity to the ways in which the researcher and the research process have shaped the collected data, including the role of prior assumptions and experience…Personal and intellectual biases need to be made plain at the outset of any research reports to enhance the credibility of the findings. The effects of personal characteristics such as age, sex, social class, and professional status…on the data collected and on the ‘distance’ between the researcher and those researched needs to be discussed.

Explicit acknowledgement of potential influences on the reviewers and research process is an important means of ensuring transparency and improving credibility of the results [21, 22]. To inform the process of reflexivity in the review and to inform understanding of how personal and professional perspectives may influence our interpretation of data, the core review team (MP, JHS, VS, RJ, SD) discussed and recorded their perspectives at the outset. These were developed through conversation and reflection on personal and professional experiences and backgrounds.

A range of perspectives was expressed by these reviewers, such as those of HCPs with backgrounds in nursing, psychology and physiotherapy, including a specialist women’s health physiotherapist and a feminist medical sociologist. Interests in holistic, biopsychosocial models of healthcare, preventative healthcare and self-management, person-centred approaches to communication and behaviour change, adherence to therapeutic exercise and health inequalities were also recorded, as well as personal experiences of UI related to pregnancy and childbirth. Two reviewers within the core team have previously published in the area of UI and maternal health, including Cochrane reviews. As it is likely that their work will be considered for inclusion in the review, the impact of closeness to the data will be acknowledged. However, these researchers will not critically appraise their own work. Authors who have not previously published in this field will provide a more distant perspective.

We will continue to reflect on personal and group perspectives and potential influences on the findings in ongoing team discussions throughout the review process. These reflections will be documented in a research report that will be available if required for any audit of our reflexive account.

### Formulating the review question

The review questions were developed collaboratively through in-depth discussion and debate between the review team members who have a range of professional healthcare and research backgrounds.

The guiding questions for our review are:

1. What are the opportunities, challenges and concerns during the childbearing years for implementation of:
  - Routine recommendation of PFME?
  - PFME education?
  - Objective assessment of PFMC and/or PFME?
  - PFMT?
  Opportunities, challenges and concerns will be investigated in relation to the following:
  (i) Organisational leadership
  (ii) Professional cultures and inter-professional working
  (iii) Individual HCPs in the course of their routine clinical practice
  (iv) Individual women during their childbearing years
2. What factors influence the acceptability and uptake of pelvic floor muscle assessment and the acceptability, uptake, adoption and maintenance of PFMT by women during their childbearing years?

The review will explore the attitudes, beliefs and behaviours of different HCPs, for example, midwives, nurse-midwives, obstetricians, physiotherapists, health care assistants, public health nurses, family doctors, antenatal and postnatal childbirth educators and ultrasonographers.

### Eligibility criteria

We intend that inclusion criteria will encompass the breadth of relevant individual, professional, inter-professional and organisational issues relating to PFME education, assessment and training during childbearing years. Consistent with the CIS method, we will conduct a purposive sample of these sources to maximise insight into these areas. As the review progresses, we may judiciously refine the inclusion criteria to ensure that the insights emerging from the review are critically incorporated.

Initial inclusion criteria will seek reports that present either a contemporary view of PFME education, assessment and training in women during childbearing years, or retrospective views of older women who have previously given birth or have been offered PFME education, assessment and training during pregnancy. Any reports from women who have never been pregnant will be excluded.

The review will identify sources that present a perspective on, report primary quantitative or qualitative data on, or report a synthesis of research about:

1. Attitudes and beliefs of women about the positive or negative physical, social and psychological consequences of pregnancy and childbirth on their pelvic floor
2. Attitudes and beliefs of HCPs about women’s understanding of their pelvic floor and how they explain the function of the pelvic floor to women
3. Views of HCPs about the impact on women’s health of PFME education, assessment and training during the childbearing years
4. Women’s and HCPs’ experiences of organisational and/or professional issues and their understanding of how these impact on PFME education, assessment and training in the childbearing years

For criteria 3 and 4, views and experiences could relate to:

- The implementation of education, assessment or training
- The acceptability of education, assessment or training
- The uptake, adoption and maintenance of education, assessment or training

Reports that are not written in the English language will be excluded.

### Information sources

At the initial stages of the review, a wide range of sources will be eligible for inclusion, including (but not limited to) editorials, opinion pieces, commentaries, comparative effectiveness studies, process evaluations, qualitative research, surveys, guidelines, professional society statements or position papers and systematic reviews. There will be no restriction on publication type. However, the scope of this review does not extend to include social media.

### Search strategy

An initial broad search strategy combining index terms and text words was developed by an information specialist to ensure that all synonyms used internationally were included (see Additional file 1). As the review focuses on women during their childbearing years, the strategy will use a set of terms describing pregnant women and mothers. This will be combined with a set of terms for PFME. Test searches and benchmarking have been conducted during development of the review protocol.

Relevant bibliographic electronic health and social science databases will be searched to identify primary research and review-level sources. These include MEDLINE, MEDLINE In Process, The Cochrane Library’s HTA, DARE, CENTRAL, EED and CDSR databases and the Centre for Reviews and Dissemination’s PROSPERO register of protocols of systematic reviews, CINAHL, EMBASE, PsycINFO, DoPHER, ASSIA, SSCI, SCI and Proquest Nursing.

To supplement the electronic searches, alternative strategies will also be used, such as searching the reference lists of included studies, backwards and forwards citation searching and applying the citation pearl growing technique to key references. A focused search of relevant Internet sites will be conducted and subject experts contacted as when required.

### Purposive sampling

In addition to the initial broad search, focused searches to identify potentially relevant material relating to topics or areas of interest which emerge as the review progresses will be run. For example, literature about midwives’ experiences of implementing practice change, or to contrast the thoughts or feelings of older women with existing UI regarding PFME with those of pregnant women. Purposive sampling will be carried out following initial analysis and in response to gaps identified following systematic searching of the literature. This sampling will also be used during later stages of the review to identify additional sources that might inform key conceptual areas. Subject experts will be contacted to identify conceptually rich sources (see Table 3 for criteria) to inform the purposive sampling strategy.

**Table 3:** Criteria used for assessing the conceptual richness of sources

‘Conceptually rich’	Explanatory but not ‘conceptually rich’	Descriptive
Theoretical concepts are unambiguous and described in sufficient depth to be useful	Consideration of the context in which the research took place	Limited or no consideration of the context in which the research took place
Relationships between and among concepts are clearly articulated	Some attempt to explain anomalous results and findings with reference to context and data	No attempt to explain anomalous results and findings with reference to context and data
Concepts sufficiently developed and defined to enable understanding *without* the reader needing to have first-hand experience of an area of practice	Correlations and relationships explained, with use of inferential statistics (quantitative studies)	Use of descriptive statistics only (quantitative studies)
Concepts grounded strongly in a cited body of literature		
Concepts are parsimonious (i.e. provide the simplest, but not over-simplified, explanation)		

### Selection process

The titles and abstracts of all papers identified by the search strategy will be screened for eligibility. Initial screening will be divided between two reviewers. Full text will be obtained for all sources that meet our initial inclusion criteria and for papers where it is unclear from the abstract whether they should be included, or where the abstract is not available. Based on initial analysis, further papers may be selected or further searches may be conducted to find material on emerging topics of interest. A sample of at least 10% at both title/abstract and full-text screening stages will be checked by a second reviewer. This approach to screening is consistent with review methods related to CIS which emphasise ongoing critical dialogue between reviewers over independent, blinded screening (for example, see the quality standards for meta-narrative review [23] and realist review [24]. Any uncertainty or ambiguity regarding inclusion of a paper will be discussed with a third reviewer. We will not measure inter-rater reliability as our focus will be on learning from these uncertainties and ambiguities and how they inform the development of the synthesis.

### Data extraction

At the initial stages of the review, ‘data extraction’ will be conducted by the review team through note-taking, annotation, discussion and conceptualisation. As the review progresses iteratively from exploration to conceptualisation to synthesis, bespoke data extraction tables will be used to extract relevant data and information from included sources.

Key characteristics of each paper will be recorded, for example, authors, publication date, date of recruitment and/or data collection (as applicable), type of paper/study design, geographical location, healthcare setting (e.g. community services, hospital), level of investigation (e.g. individual, professional, organisational/institutional), research methods (if applicable) and theoretical perspective or approach. Study findings will be recorded at the level of the primary study authors’ analysis.

A sample of completed data extraction forms completed by each reviewer will be checked by another reviewer for accuracy in relation to data extraction fields which involve quantitative data or key information, such as study design, and for completeness relating to the extraction of relevant qualitative data. The data extraction process itself will involve critical discussion between reviewers and the wider team so that data are not simply ‘classified’ but are used to begin to develop a line of argument that feeds into the final synthesis and synthesising argument.

### Critical appraisal

Articles will be prioritised according to relevance to the review question. Papers will not be excluded on the basis of quality but will be judged on credibility and contribution as part of the synthesis process. Critical appraisal of research will be informed by the Wallace et al. (2004) criteria [25] (see Additional file 2). These criteria cover key research quality components of rigour (e.g. sampling, data collection and data analysis) relevant across different fields of practice. Using these criteria will also enable identification of whether or not certain aspects of a study have particular strengths or weaknesses. This will enable critical consideration of relevance and rigour together and enable the review team to be explicit about the reasons for how evidence from different sources has been used.

### Data synthesis

The data analysis and synthesis approach used in CIS is similar to that of primary qualitative research. Data analysis will involve the following steps [17]:

1. *Detailed reading and inspection of included papers* will enable familiarisation with the evidence and identification of recurring themes and concepts. These will be constantly compared between papers to identify connections and relationships between themes. This process will develop a critique of the evidence, allowing constructs to be developed from original concepts in the papers and through generation of new synthetic constructs. An ongoing critique of the evidence will seek to question assumptions, arguments and interpretations presented in the papers. Constructs and critique will be combined to generate a synthesising argument to illustrate links and relationships between existing and synthetic constructs in the literature. This theoretical framework will guide further selection of potentially relevant literature.
2. *A purposive sample of sources* will be drawn upon to:
  Refine already well-developed concepts specifically in relation to the research questions
  Build and refine weaker concepts
  Critically engage across a diverse body of literature, for example, to inform alternative framings of issues and potentially build new concepts
  Purposive sampling will be carried out following initial systematic searching of the literature and during later stages of the review to identify additional sources that might inform key conceptual areas. Subject experts will be contacted to identify conceptually rich sources (Table 3) to inform the purposive sampling strategy.
3. *Data will be managed using appropriate software, where studies will be coded to identify patterns and themes* across all data. Data may be combined across studies and transformed into new explanatory themes to allow re-interpretation of the findings within the context of the evidence as a whole.
4. *New themes and original constructs reported in the primary research reports will be integrated into the theoretical framework* and constantly compared against data in the original research reports. The developing framework will enable links and relationships between the theoretical constructs to be identified. To help structure our inquiry into how professionals in complex organizational environments engage with, implement and sustain novel interventions, we will use May’s extended Normalization Process Theory (NPT) [26, 27] to provide an initial framework for the organisation and analysis of implementation issues. We will also interpret findings in relation to relevant literature, such as how to understand individuals’ attitudes and beliefs (e.g. Leventhal’s ‘Common Sense Model’ [28]) and how individuals’ attitudes and beliefs relate to achieving behaviour change (e.g. Michie et al.’s COM-B (Capability, Opportunity, Motivation–Behaviour) model [29]; the Theoretical Domains Framework [30]). The synthesis will pay particular attention to potential inter-relationships between factors associated with health inequalities (e.g. socio-economic status, ethnicity) and the opportunities, challenges and concerns regarding the implementation, acceptability, uptake, adoption and maintenance of PFME education, assessment and training.
5. *The framework will be used to develop a conceptual model* that maps the relationships between organisational leadership, professional cultures and inter-professional working, individual HCPs in the course of their routine clinical practice, individual women during their childbearing years and the effective implementation of PFME education, assessment and training.

See Additional file 3 for a completed PRISMA-P+ checklist for this review protocol.

## Discussion

This protocol was developed through an extensive collaborative process between an interdisciplinary research team. An ongoing iterative process of collaboration, discussion and refinement of the theoretical framework will ensure that the conceptual model produced by the CIS provides a useful and useable framework for subsequent APPEAL studies and future intervention development and evaluation. This will be achieved through collation of meaningful examples, either empirical or hypothetical, that illustrate the relationships expressed in the conceptual model, and identification of potential links between the conceptual model and drivers for change. For example, the model may identify potential mediating or moderating variables that could explain or affect the strength of relationship between independent and dependent variables that will inform future intervention development and testing.

Using CIS for this review will enable synthesis of a large and diverse range of literature, including primary qualitative and quantitative research, review-level research and non-empirical evidence [17, 31], resulting in a broad perspective on PFME to be incorporated into the conceptual model. A key advantage of CIS is that it provides a critical view of existing evidence so that new insights and perspectives can be developed.

The findings from the review will provide generalisable evidence and context awareness [18] relating to organisational, professional and individual opportunities, challenges and concerns that will inform planning, implementation and evaluation of the APPEAL intervention.

### Dissemination

The review will be feeding into the wider APPEAL research project to inform the development and evaluation of the training package for midwives. Dissemination of the review report is also likely to include presentation at local, national and/or international research meetings and publication in an appropriate peer-reviewed journal.

## Additional files

Additional file 1: Search strategy. (DOCX 15 kb)
Additional file 2: Criteria for quality appraisal. (DOCX 15 kb)
Additional file 3: Completed PRISMA-P+ checklist. (DOCX 29 kb)

## Abbreviations

APPEAL: Antenatal Preventative Pelvic floor Exercises And Localisation
CIS: Critical interpretive synthesis
HCP: Healthcare professional
NICE: National Institute for Health and Care Excellence
PFM: Pelvic floor muscle
PFMC: Pelvic floor muscle contraction
PFME: Pelvic floor muscle exercise
PFMT: Pelvic floor muscle training
UI: Urinary incontinence
