# Supplementary material for: Opportunities, challenges and concerns for the implementation and uptake of pelvic floor muscle assessment and exercises during the childbearing years: protocol for a critical interpretive synthesis
Source: Syst Rev. 2017 Jan 25;6:18. doi: 10.1186/s13643-017-0420-z (PMC5267404; doi:10.1186/s13643-017-0420-z)
Supplement: Additional file 1: — Search strategy. (DOCX 15 kb) [file 13643_2017_420_MOESM1_ESM.docx]

# Additional file 1

Search strategy.

Database: Ovid MEDLINE(R) 1946 to June Week 3 2016

Search Strategy:

1 exp Pelvic Floor/

2 muscle contraction/

3 (pelvic floor adj2 muscle$).ti,ab.

4 ((Pelvic floor or pelvic muscle) adj3 (strength$ or therapy or function$ or dysfunction$ or self-efficac$ or move$ or mobility or lift$ or endurance or confiden$ or thickness or train$ or exercis$ or rehabilitat$ or contract$ or educat$)).ti,ab.

5 (PFM$ or PFE$).mp. or kegel.ti,ab.

6 1 and 2

7 3 or 4 or 5 or 6

8 (pregnan$ or expectant or expecting or antenatal$ or prenatal$ or ante-natal$ or pre-natal$ or postnatal$ or postpartum or post-partum or post-natal or puerper$ or perinatal$ or peri-natal$ or maternal$ or maternity or mother$ or childbirth or child-bearing).ti,ab.

9 Mothers/

10 exp Pregnancy/

11 exp Parturition/

12 childbirth.ti,ab.

13 or/8-12

14 7 and 13

15 limit 14 to (english language)
