# Supplementary material for: Opportunities, challenges and concerns for the implementation and uptake of pelvic floor muscle assessment and exercises during the childbearing years: protocol for a critical interpretive synthesis
Source: Syst Rev. 2017 Jan 25;6:18. doi: 10.1186/s13643-017-0420-z (PMC5267404; doi:10.1186/s13643-017-0420-z)
Supplement: Additional file 2: — Criteria for quality appraisal. (DOCX 15 kb) [file 13643_2017_420_MOESM2_ESM.docx]

# Additional file 2

Criteria for quality appraisal

| 1 | Question | Is the research question clear? |
| --- | --- | --- |
| 2 | Theoretical perspective | Is the theoretical or ideological perspective of the author (or funder) explicit?  Has this influenced the study design, methods, or research findings? |
| 3 | Study design | Is the study design appropriate to answer the question? |
| 4 | Context | Is the context or setting adequately described with regard to implementation? |
| 5 | Sampling | Is the sample adequate to explore the range of subjects and settings?  Has it been drawn from an appropriate population? |
| 6 | Data collection | Was the data collection adequately described?  Was it rigorously conducted to ensure confidence in the findings? |
| 7 | Data analysis | Was there evidence that the data analysis was rigorously conducted to ensure confidence in the findings? |
| 8 | Reflexivity | Are the findings substantiated by the data and has consideration been given to any limitations of the methods or data that may have affected the results? |
| 9 | Generalisability | Do any claims to generalisability follow logically and theoretically from the data? |
| 10 | Ethics | Have ethical issues been addressed and confidentiality respected? |

From Wallace A, Croucher K, Quilgars D, Baldwin S. Meeting the challenge: developing systematic reviewing in social policy. Policy and Politics 2004; 32:455-470.
